# Copper-Catalyzed Difluoroalkylation Reaction

**DOI:** 10.3390/molecules27238461

**Published:** 2022-12-02

**Authors:** Dao-Qing Dong, Shao-Hui Yang, Pei Wu, Jin-Zhi Wang, Ling-Hao Min, Hao Yang, Meng-Yu Zhou, Ze-Hui Wei, Cai-Zhen Ding, Yan-Li Wang, Jia-Hui Gao, Shu-Jie Wang, Zu-Li Wang

**Affiliations:** 1 College of Chemistry and Pharmaceutical Sciences, Qingdao Agricultural University, Qingdao 266109, China; 2 Shandong Academy of Pesticide Sciences, Beiyuan Street, Jinan 250033, China; 3 Tancheng County Agricultural Technology Popularization Center, Linyi 276100, China; 4 Qingdao Zhongda Agritech Co., Ltd., Qingdao 266109, China; 5 National Engineering Research Center of Low-Carbon Processing and Utilization of Forest Biomass, Nanjing Forestry University, Nanjing 210037, China

**Keywords:** difluoroalkylation, copper, radical, cyclization, multicomponent, coupling reaction

## Abstract

This review describes recent advances in copper-catalyzed difluoroalkylation reactions. The RCF_2_ radical is generally proposed in the mechanism of these reactions. At present, various types of copper-catalyzed difluoroalkylation reactions have been realized. According to their characteristics, we classify these difluoroalkylation reactions into three types.

## 1. Introduction

**Figure 1:**
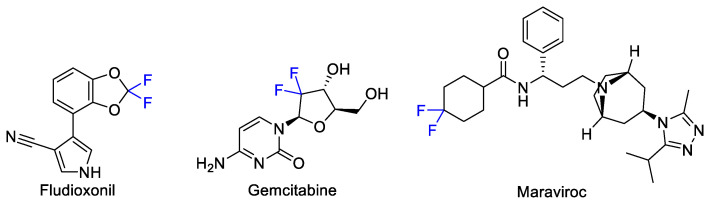
Difluoroalkylated drugs or candidates.

**Figure 2:**
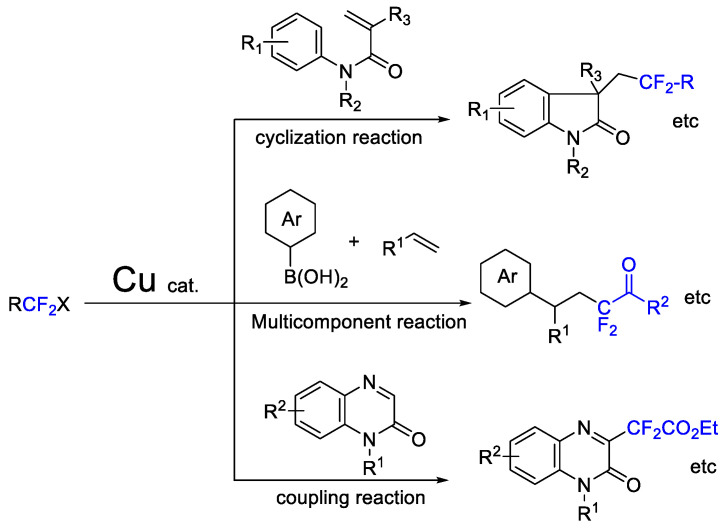
Copper-catalyzed difluoroalkylation reactions.

Difluorinated compounds such as fludioxonil [1], gemcitabine [2] and maraviroc [3] play an important role in the agrochemical, pharmaceutical, and materials science industries due to their special physical and chemical properties (Figure 1) [4,5,6]. As a consequence, more and more attention has been paid to the development of simple and efficient methods for introducing difluoroalkyl groups in recent years. Transition-metal-catalyzed difluoroalkylation via cross-coupling is an efficient and attractive method for this reaction. Various catalysts (Pd, [7] Ir, [8,9] Cu [10], Ni [11], etc. [12,13]) have been successfully applied in difluoroalkylation reactions [14,15,16,17,18,19]. However, it is still a great challenge to selectively control the catalytic cycle and obtain the desired fluorinated compounds by directly using classical transition-metal-catalyzed cross-coupling reactions, as some difluoroalkyl metal species have significantly different properties from their nonfluorinated analogues. They are unstable and can easily protonate, dimerize, and/or produce other unknown byproducts. Compared with other metal catalysts, copper has the advantages of low cost, low toxicity, wide availability, etc. Accordingly, copper-catalyzed reactions have seen much progress [20,21,22,23,24,25,26,27,28,29,30]. As early as 1986, a copper-mediated Ullmann cross-coupling reaction of halodifluoroacetates with activated (hetero)aryl electrophiles was reported by the Kobayashi group [10]. However, excess copper was required in this system. With regard to copper-catalyzed difluoroalkylation reactions, there have been several reports in recent years. In this review, the progress of difluoroalkylation in the last 3 years is summarized. According to their differences, these difluoroalkylation reactions were classified into three types (Figure 2).

## 2. Difluoroalkylation–Cyclization Reactions

**Scheme 1:**
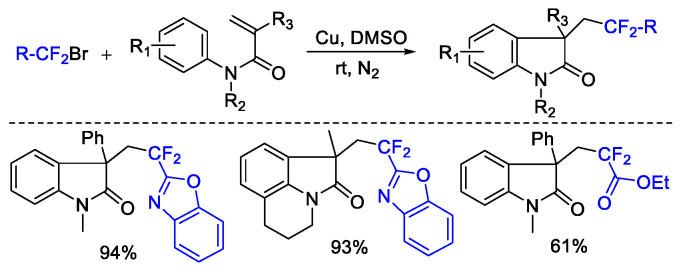
The copper-catalyzed difluoromethylenation of N-arylacrylamides.

Jiang’s group reported the copper-mediated difluoromethylenation between N-arylacrylamides and benzo-1,3-oxazolic difluoromethylbromide (Scheme 1) [31]. Control experiments were conducted, implying that a radical intermediate was possibly involved in this transformation. RCF_2_ radicals, which were generated from the Cu(0)-mediated single-electron transformation with RCF_2_Br, could be added to the terminal end of the C=C double bond of N-arylacrylamide. Subsequently, an intramolecular radical cyclization reaction occurred to form the final product.

**Scheme 2:**
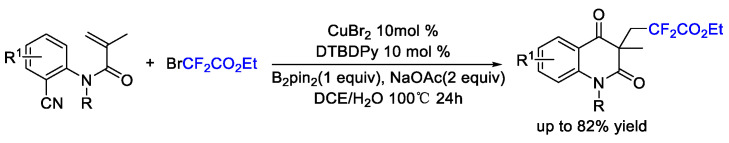
Difluorinated quinoline-2,4-diones catalyzed by copper.

In 2018, Shi’s group established a facile method to construct diverse difluorinated quinoline-2,4-diones via the Cu-catalyzed direct difluoromethylation of activated alkenes. A difluoromethyl radical addition/cyclization reaction mechanism was indicated for this strategy (Scheme 2) [32]. It is worth noting that this reaction could also be carried out in the presence of a visible-light photo-redox catalysis.

**Scheme 3:**
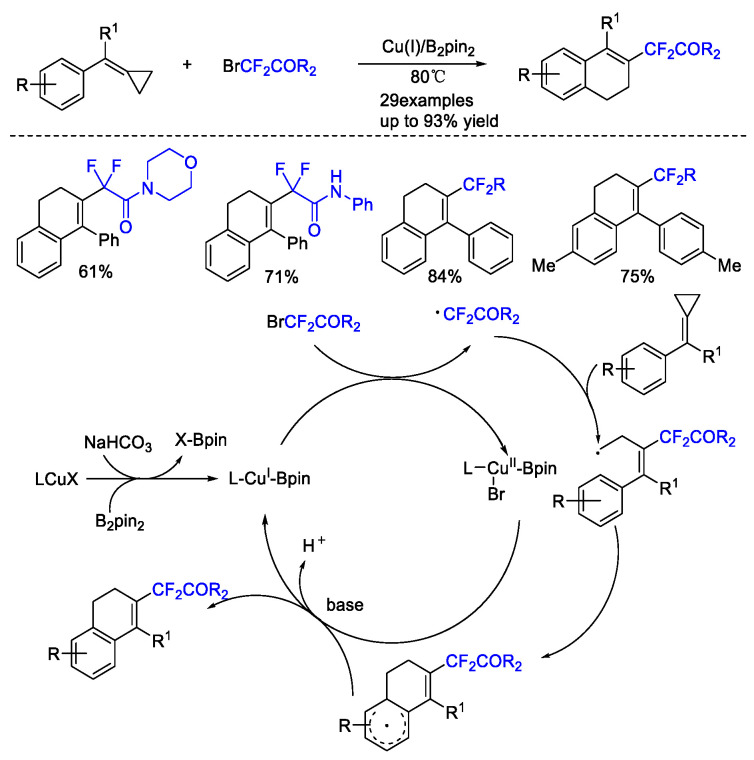
The copper/B_2_pin_2_-catalyzed difluoroalkylation of methylenecyclopropanes.

The copper/B_2_pin_2_-mediated difluoroalkylation of methylenecyclopropanes with bromodifluorinated acetates and acetamides was illustrated by Wang and coworkers (Scheme 3) [33]. Various substrates reacted smoothly in this reaction system. Both the catalyst and an inert atmosphere are necessary for the successful operation of the reaction. A Cu(II)/Cu(I) catalyzed tandem radical process, which involved ring-opening/intramolecular cyclization, was proposed in the reaction mechanism.

**Scheme 4:**
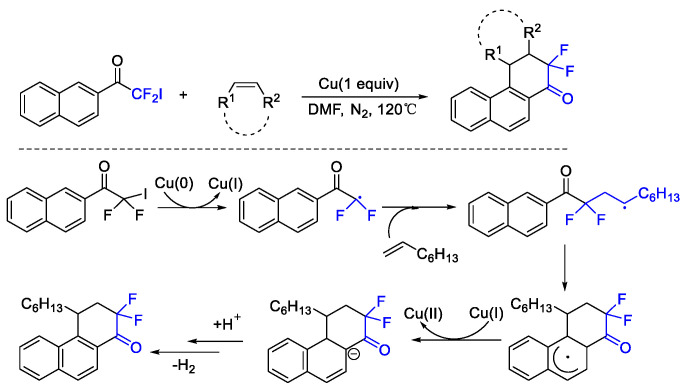
The copper-mediated radical cyclization of naphthalenyl iododifluoromethyl ketones.

Difluoroacyl heterocyclic compounds are widely found in various bioactive compounds and agricultural chemicals [34,35]. At present, these reactions have been studied extensively [36,37]. In 2019, Wu’s group realized the copper-mediated free-radical cyclization of naphthalenyl iododifluoromethyl ketones with olefins (Scheme 4) [38]. The corresponding difluoroacyl compounds with moderate yields were obtained. Mechanistic investigations suggested that difluorinated radical intermediates, which were generated from the reduction reaction of RCF_2_I with Cu(0), were involved in the reaction pathway. 

**Scheme 5:**
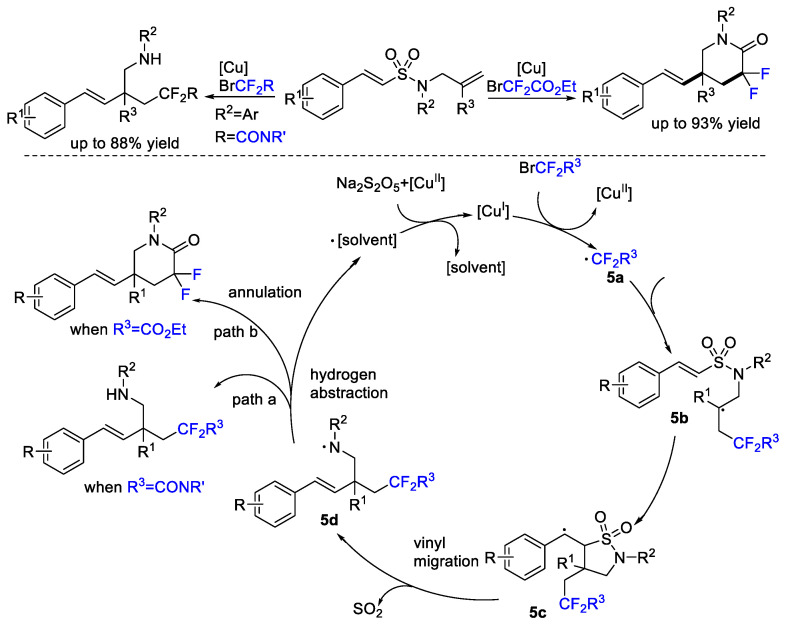
The difunctionalization of unactivated alkenes through desulfonylation.

Wang’s group reported, for the first time, the difunctionalization of unactivated alkenes through desulfonylation-initiated distal alkenyl migration. Using this method, previously unknown 3,3-difluoro-5-styrylpiperidin-2-one derivatives with a quaternary stereocenter can be efficiently constructed (Scheme 5) [39]. A radical mechanism was involved in this reaction. Initially, a single-electron transfer process occurs between the Cu(I) species and BrCF_2_R_3_ to generate fluoroalkyl radical 5a and Cu(II) species. The less steric terminal olefin is then selectively attacked by fluoroalkyl radical 5a, yielding a transient alkyl radical 5b. Radical 5b reacts with the internal double bond to form cyclized radical 5c. Then, radical 5c undergoes the desulfonylation reaction, forming the critical N-center radical 5d. Finally, the 3, 3-difluoro-5-pyranopridine-2-ketone product was produced via the cyclization reaction.

**Scheme 6:**
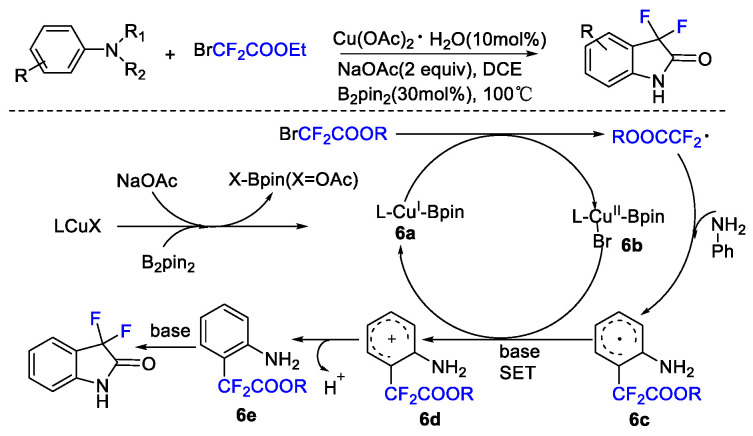
The copper/B_2_pin_2_-catalyzed C–H difluoroacetylation–cycloamidation of anilines.

Song’s group developed an effective method for the synthesis of 3,3-difluoro-2- oxindoles by copper/B_2_pin_2_-catalyzed C-H difluoroacetylation–cycloamidation of aniline with ethyl bromodifluoroacetate. (Scheme 6) [40]. In this strategy, amino groups act as directing groups to regioselectively provide ortho-difluoroacetylated products. In the presence of a base, LCu(I)-Bpin species 6a was formed by the reaction of LCuX(I) and B_2_pin_2_. Then, species **6a** reduced BrCF_2_CO_2_R to form the CF_2_CO_2_R radical. Subsequently, the CF_2_CO_2_R radical reacted with aniline to produce an amino-oriented intermediate 6c. Finally, the desired products, 3,3-difluoro-2- oxindole derivatives, were isolated via the SET pathway and an intermolecular cycloamidation reaction of ester with the amino group.

**Scheme 7:**
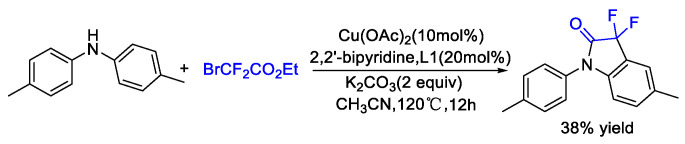
The copper-catalyzed annulation of N-substituted anilines.

The difluoromethylation reaction using aryl-substituted anilines as substrates is challenging due to the steric disadvantage, as well as due to entropy. The C–H [3 + 2] annulation of N-aryl or alkyl-substituted anilines with bromodifluoroacetate catalyzed by copper was realized by Li’s group (Scheme 7) [41]. The corresponding products, 3,3′-disubstituted oxindoles, can be obtained with moderate to good yields.

**Scheme 8:**
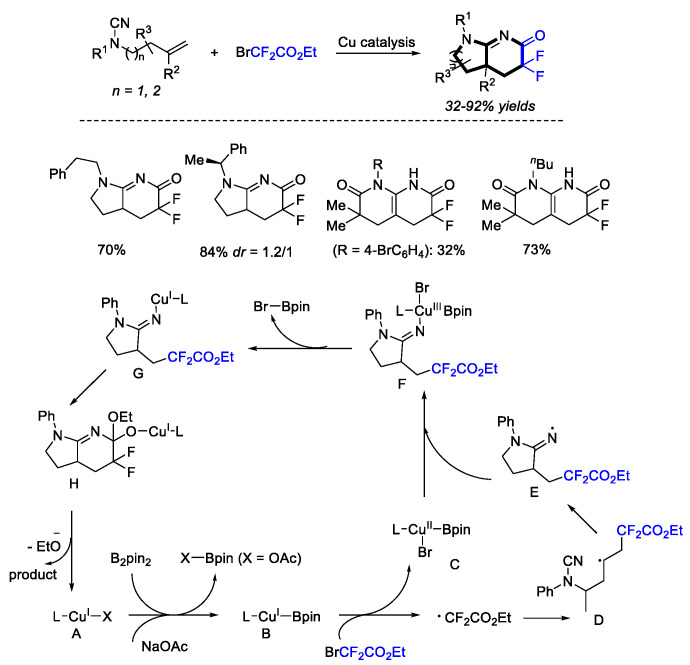
The difluoroalkylation of an olefin/nitrile insertion/cyclization catalyzed by copper.

Difluoroalkylation of an olefin/nitrile insertion/cyclization tandem sequence of N-cyanamide olefins catalyzed by copper was realized by Liao’s group, which provides a convenient synthesis method for obtaining difluorobicycloamides containing imine groups in a sustainable manner (Scheme 8) [42]. The protocol has the advantages of a high yield, wide substrate range and good functional group compatibility. In addition, diverse synthetic transformations have also been demonstrated for preparing various functionalized difluorinated aza-heterocycles.

**Scheme 9:**
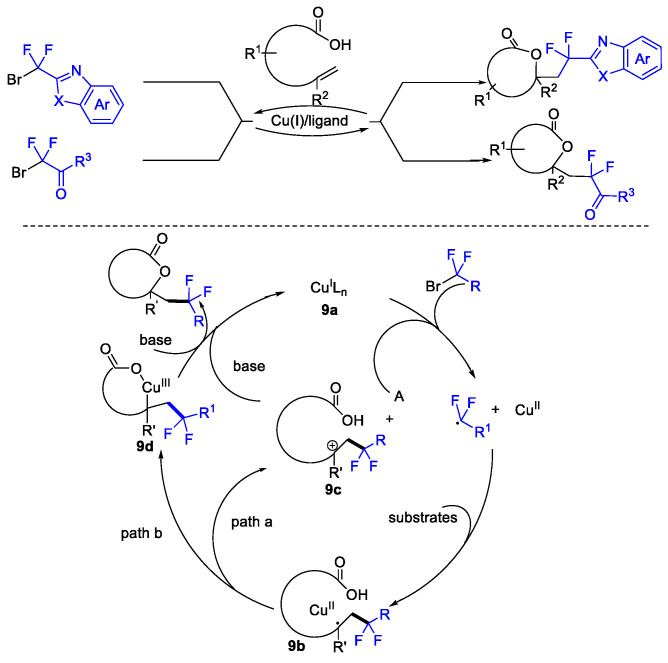
The cyclization between alkenyl carboxylic acids and difluoroalkyl reagents.

**Scheme 10:**
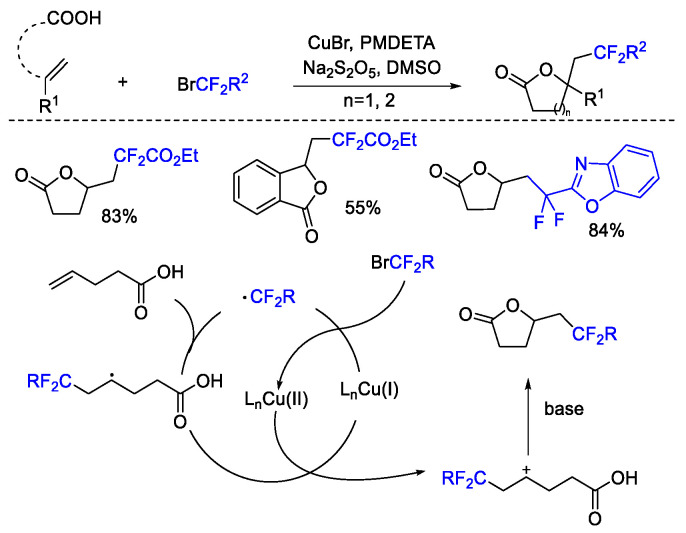
The copper-catalyzed cyclization of unsaturated carboxylic acids.

In recent years, the cyclization reaction of alkenyl carboxylic acids with difluoroalkyl reagents has been established for the synthesis of difluoroalkyl lactones. In 2018, Li’s group achieved difluoroalkylation of alkenyl carboxylic acids catalyzed by a copper catalyst under mild reaction conditions (Scheme 9) [43]. The high-value lactones were delivered containing CF_2_ with moderate to excellent yields. A catalytic reaction pathway involving free radicals was proposed. Firstly, the RCF_2_ radical and Cu(II) species were generated from an initial single-electron transfer (SET) from Cu(I)Ln 9a to RCF_2_Br. Subsequently, the difluoroalkyl radical intermediate 9b was obtained from the addition reaction of the RCF_2_ radical with unsaturated carboxylic acid. Then, 9b was oxidized by Cu(II) to give the carbocation intermediate 9c, and the copper(I) catalyst was regenerated. Finally, an intramolecular nucleophilic attack reaction could provide the final product (Scheme 36, path a). Another pathway that forms the final product under basic conditions through the reductive elimination of Cu(III) intermediate 9d is also possible (Scheme 9, path b). Another copper-catalyzed difluoroalkylation between alkenyl carboxylic acids and BrCF2R reagents for the synthesis of lactone was also demonstrated by Wang and coworkers (Scheme 10) [44]. A similar reaction mechanism which contains a Cu(I)/Cu(II) cycle was suggested for this transformation.

**Scheme 11:**
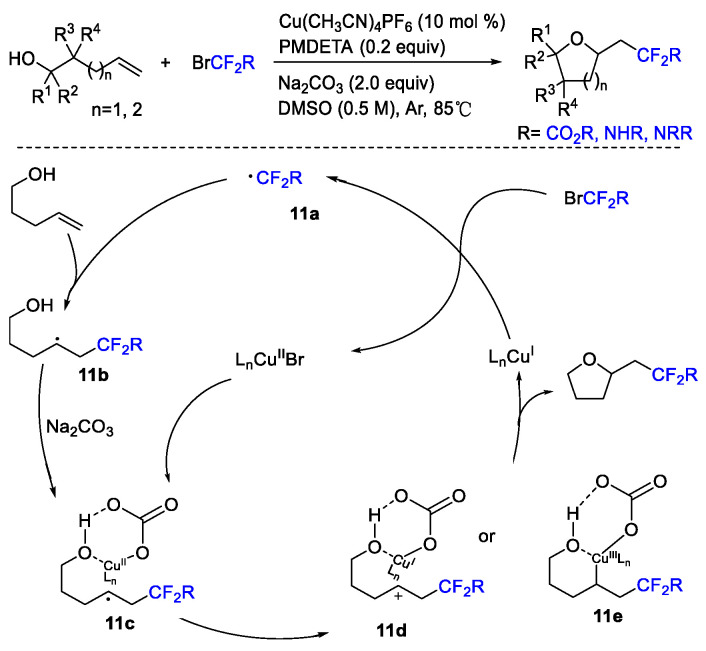
The intramolecular oxydifluoroalkylation of hydroxyl-containing alkenes catalyzed by copper.

**Scheme 12:**
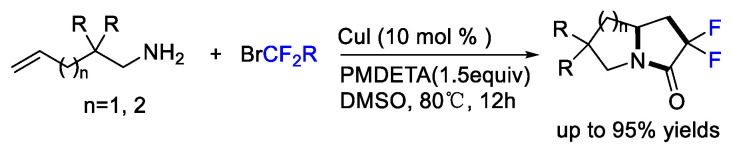
The copper-catalyzed reaction for synthesis of difluorinated polycycles.

Wang and Guo’s group established a copper-catalyzed intramolecular oxydifluoroalkylation of hydroxyl-containing alkenes and various fluoroalkylated tetrahydrofurans observed in this reaction [45]. In addition to ethyl bromodifluoroacetate, other functionalized difluoroalkylated bromides also reacted smoothly in this system. The reaction mechanism is depicted in Scheme 11. First, the oxidation between Cu(I) and ethyl bromodifluoroacetate occurred to produce the RCF_2_ radical 11a and Cu(II) intermediate. Intermediate 11b could be formed via the addition reaction between radical 11a and alkenes, which was captured by the Cu(II) species with the help of the Na_2_CO_3_ base to form intermediate 11c. Then, two possible intermediates, 11d or 11e, can be formed. Intermediate 11d can undergo an activated-alcohol nucleophilic attack on carbon cation species and generate the desired product. Alternatively, reductive elimination of intermediate 11e can also provide the final product. Both of the two pathways are possible at the moment. In 2019, this group demonstrated that a variety of difluorinated nitrogen-containing polycyclic compounds can be obtained when amine-containing olefins are used instead of hydroxy-containing olefins as substrates (Scheme 12) [46]. The electrophilic RCF_2_ radical and Cu(II) intermediates produced from the oxidation of Cu(I) species by BrCF_2_CO_2_Et via single-electron transfer (SET) were also described in a possible reaction mechanism. Recently, Luo’s group also realized the photoredox-induced oxydifluoroalkylative cyclization of alkenes using R_f_I as substrates [47].

**Scheme 13:**
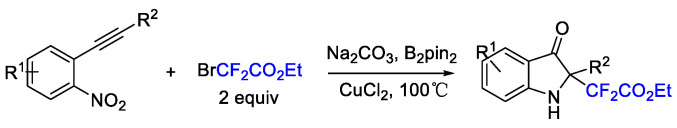
The copper-catalyzed redox cycloisomerization.

Copper-catalyzed redox cycloisomerization between non-prefunctionalized nitroalkynes and BrCF_2_CO_2_Et for the synthesis of C2-tetrasubstituted indolin-3-ones was achieved by Song’s group (Scheme 13) [48]. Using diboron as the reducing reagent, this reaction can be carried out using a one-pot protocol, and a fluorine-containing noncarbon quaternary center can be constructed. The author conducted the experiment for mechanism research. The results showed that B_2_pin_2_ captures an oxygen in NO_2_ with the help of Na_2_CO_3_. The other oxygen in NO_2_ was converted into a C=O bond of C2-tetrasubstituted indolin-3-ones.

**Scheme 14:**
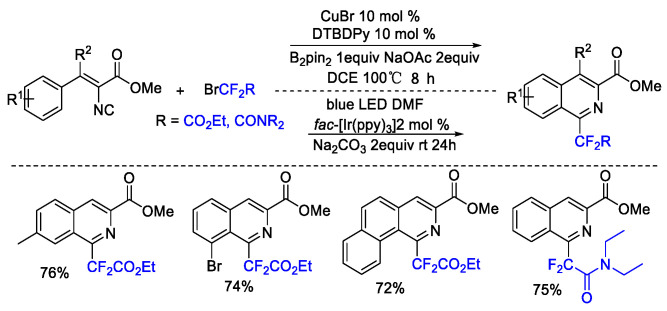
The Cu/B_2_pin_2_-promoted cascade cyclization of vinyl isocyanides.

Shi’s group described the cyclization reaction of vinyl isocyanides with bromodifluoroacetic derivatives promoted by the Cu/B_2_pin_2_ system, and various 1-difluoroalkylated isoquinolines were generated (Scheme 14) [49]. Preliminary mechanistic studies were conducted and the results implied that a tandem radical cyclization process was possible for this reaction. In addition, visible light could also promote this transformation efficiently.

**Scheme 15:**
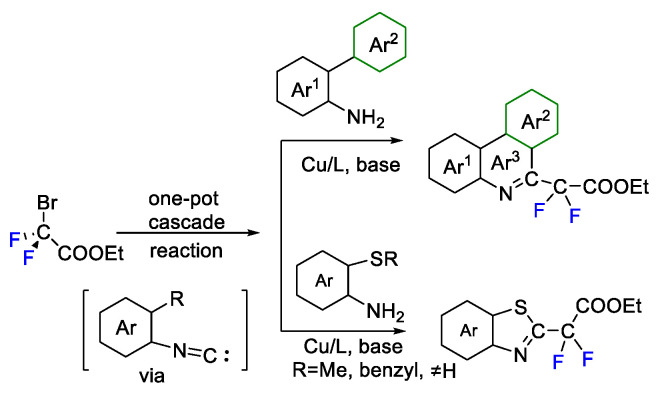
The reaction of BrCF_2_COOEt with primary amines.

In 2018, BrCF_2_CO_2_Et was discovered by Song’s research group to play a dual role as a difluoroalkylation reagent and C1 synthon in the reaction of BrCF_2_CO_2_Et with a primary amine (Scheme 15) [50]. BrCF_2_CO_2_Et reacts with Na_2_CO_3_ through decarboxylation and debromination to form difluorocarbene, which can participate in the catalytic cycle. Based on DFT calculations and experimental observations, they found that the base plays an important role in the reaction of primary amines and difluorocarbene to form the key intermediate isocyanides. 

**Scheme 16:**
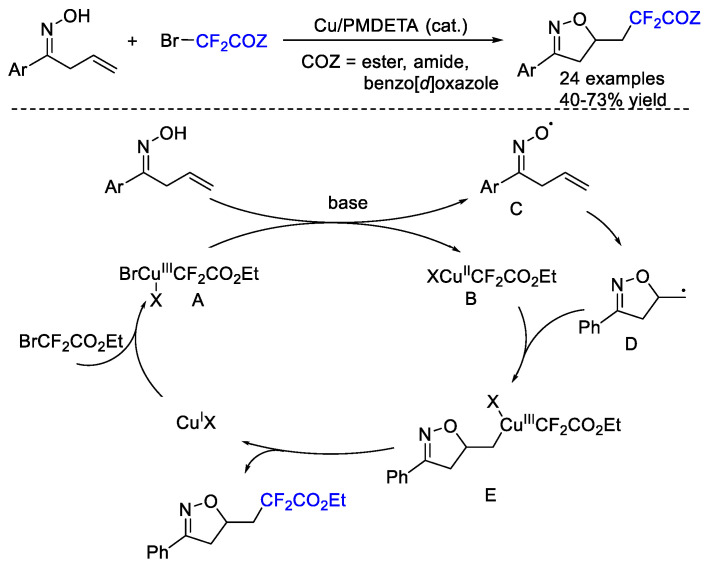
The oxydifluoroalkylation of unsaturated oximes.

The copper-catalyzed oxydifluoroalkylation of β, γ-unsaturated oximes was studied by Wang and coworkers (Scheme 16) [51]. A Cu(III)/Cu(I) catalytic cycle was described for this transformation. The reaction proceeds through a difluoroalkylation cascade of olefins, followed by a nucleophilic reaction of the hydroxyl group of the oxime. This method has the advantages of mild reaction conditions, low catalyst cost and wide substrate range, which makes it a simple method for the preparation of the fluorine-containing side-chain isoxazoline. 

**Scheme 17:**
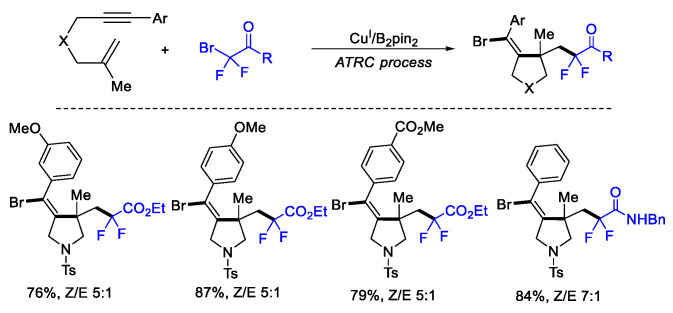
The copper-catalyzed bromodifluoroacetylation cyclization reaction.

Zhu’s group reported on the copper-catalyzed bromodifluoroacetylation cyclization reaction (Scheme 17) [52]. The treatment of bromodifluoroacetic acid derivatives with Cu(I) and B_2_Pin_2_ can generate difluoroalkyl radicals and trigger radical addition/cyclization/bromination sequences. Bromodifluoroacetyl-derived esters, amides, and ketones are well compatible in this system, generating various vinyl C–bromine bonds containing functionalized heterocycles with high yields. Two possible alternative bromination processes are proposed for the C-Br bond formation step in the vinyl intermediate: a possible radical chain-reaction mechanism and an organic metal Cu(III)/Cu(I) process.

**Scheme 18:**
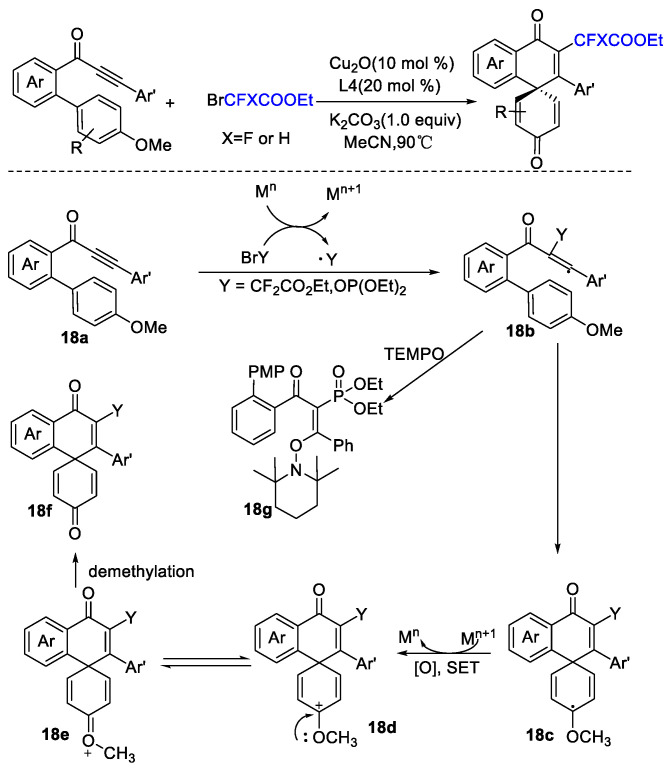
The copper-catalyzed spirocyclization of biaryl ynones.

Zhu’s group was the first to achieve the copper-catalyzed cascade radical addition/dearomative spirocyclization of biaryl ynones for the preparation of difluoromethylated spiro [5.5] trienones (Scheme 18) [53]. A series of spiro compounds can be constructed via this reaction. Monofluoromethylated and phosphorated spiro [5.5] trienones were also produced when diethylphosphite was used as a substrate in the presence of Ag as a catalyst. A fluoroalkyl radical was generated in the reaction catalytic cycle. The addition of the RCF_2_ radical to the C-C triple bond of 18a produced the vinyl radical 18b, which was followed by 6-exo-trig cyclization forming intermediate 18c, and the oxidation reaction of 18c by M^n+1^ could produce oxocarbenium ion 18d. Finally, the desired product was formed via a demethylation reaction.

**Scheme 19:**
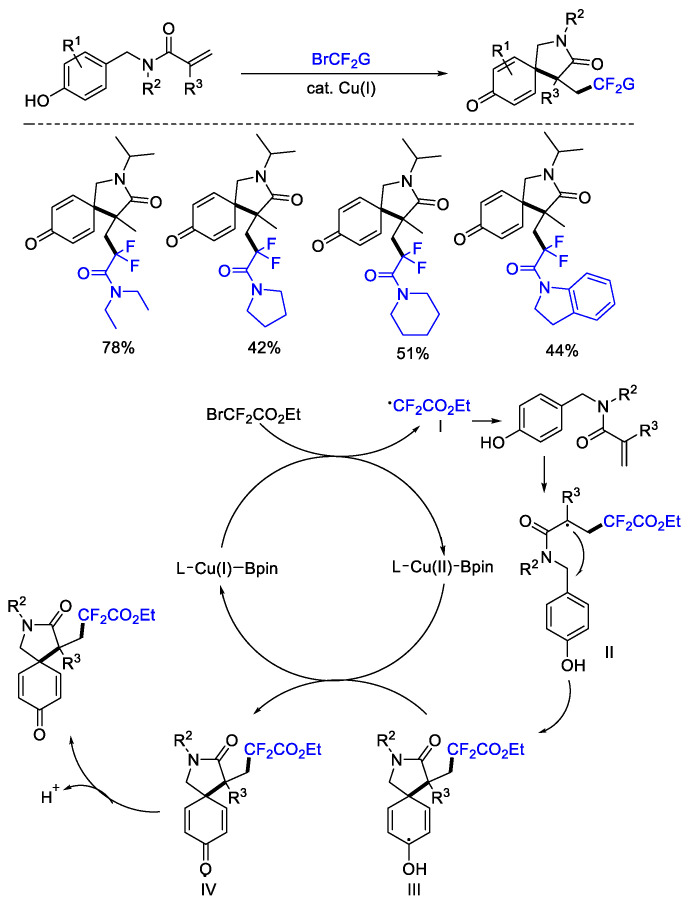
The synthesis of difluoroalkyl 2-azaspiro [4.5] decane.

An effective method for the preparation of difluoroalkyl 2-azaspiro [4.5] decane by copper-catalyzed difluoroalkylation of N-benzylacrylamide with ethyl bromodifluoroacetate was developed by Han’s group (Scheme 19) [54]. The difluoroalkylation of substituted N-benzylacrylamides, 5-exo cyclization, and dearomatization reactions were suggested in the cascade reaction. In addition, the obtained product can be successfully converted into saturated spirocyclohexanone scaffolds and difluoroalkyl quinolinone.

## 3. Difluoroalkylation–Multicomponent Reactions

**Scheme 20:**
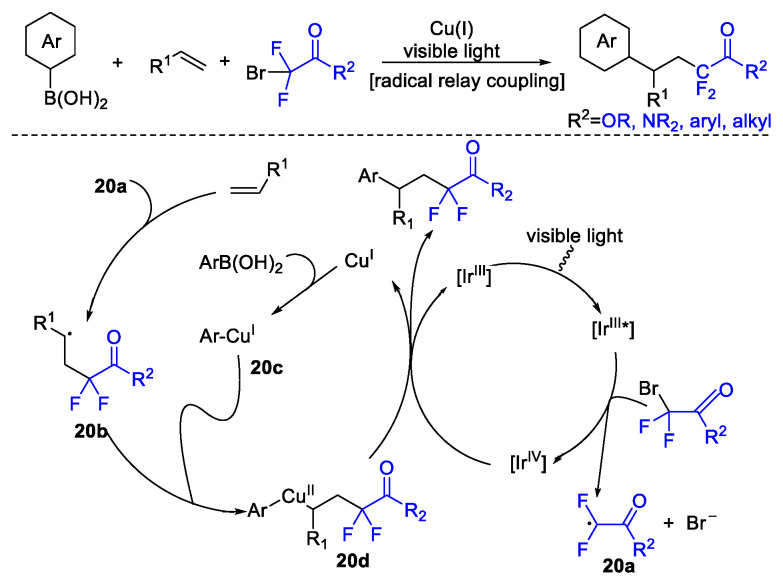
The synthesis of γ-arylation of carbonyl compounds.

Alkenes were found to react well with fluoroalkyl halides and boronic acids, and the γ-arylation of carbonyl compounds can be realized via these reactions. Shu and coworkers found that the copper and visible-light catalysis plays an important role in this reaction [55]. The reaction mechanism is described in Scheme 20. The oxidative quenching reaction between the excited Ir*(III) photocatalyst and BrCF_2_CO_2_Et occurred to form radical intermediate 20a and the oxidized photocatalyst Ir(IV). Then, the addition reaction of 20a and alkenes could provide a new alkyl radical intermediate 20b. With the help of a base, the reaction between Cu(I) and aryl boronic acid occurred to form the arylcopper intermediate 20c, which could be converted to 20d via recombination with 20b. Then, the final product was generated from 20d via reductive elimination, with the concurrent liberation of Cu(I) and Ir(III) species.

**Scheme 21:**
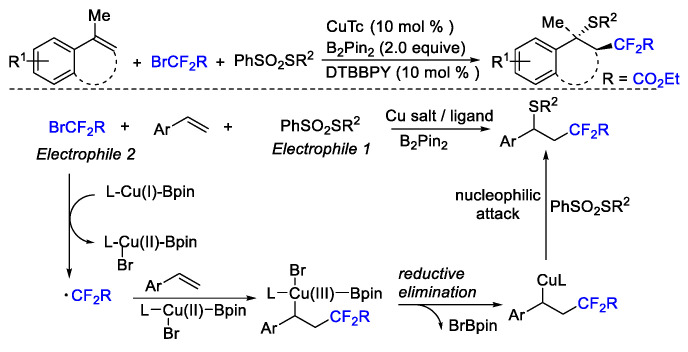
The Truce–Smiles rearrangement.

Song’s group found that the difluoroalkylation–thiolation of aryl alkenes could efficiently proceed in the presence of copper/B_2_pin_2_ (Scheme 21) [56]. As an organic reducing reagent, B_2_pin_2_ plays an important role in this reaction process, enabling the simultaneous formation of C (sp3)-C (F_2_R) and C (sp3)-S (R) bonds by using two electrophilic substrates. Radical trapping experiments were conducted, and the results implied that the CF_2_CO_2_Et radical was involved in the catalytic cycle.

**Scheme 22:**
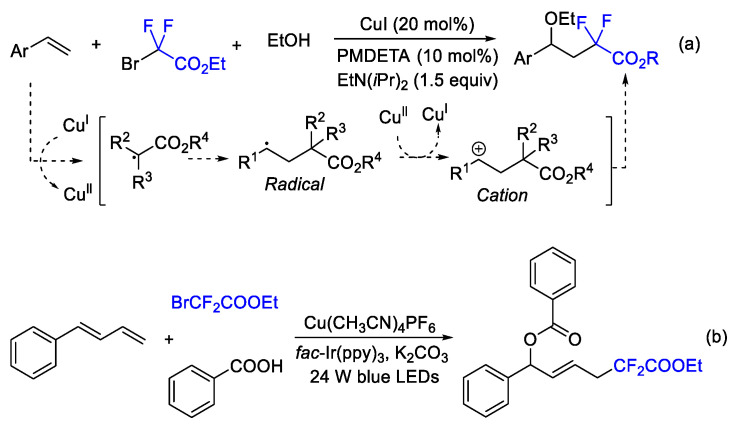
(**a**)The three-component difluoroalkylation of alkenes with BrCF_2_CO_2_Et and alcohol. (**b**) The three-component 1,4-difluoroalkylesterification of 1-aryl-1,3-dienes with BrCF_2_CO_2_Et and acids.

In 2019, the iron-mediated difluoroalkylation–thiolation of alkenes with BrCF_2_CO_2_Et and RSH was realized by Cai and coworkers [57]. This reaction enabled the simultaneous formation of Csp3–Csp3 and Csp3–S bonds. Thiols can be activated by FeCl_2_ via the Fe/S complex [58]. In the same year, the same group [59] described the photocatalyzed three-component difluoroalkylamination of alkenes using BrCF_2_CO_2_Et and amines. Besides amines and thiols, the Nishikata group found that alcohol was also a good substrate for this type of reaction (Scheme 22a) [60]. A radical and cation crossover mechanism mediated by copper was proposed as the reaction mechanism. Chen and coworkers achieved the selective three-component 1,4-difluoroalkylesterification of 1-aryl-1,3-dienes mediated by a dual copper and photoredox catalysis (Scheme 22b) [61]. This reaction provided a new method for the synthesis of difluoroalkylated allylic esters. The intramolecular two-component 1,4-difluoroalkylesterification of 1-(1,3-butadienyl) benzoic acids for the synthesis of 3-substituted benzobutyrolactones was also smoothly realized. A radical mechanism was possible for this transformation based on the results of mechanistic studies.

**Scheme 23:**
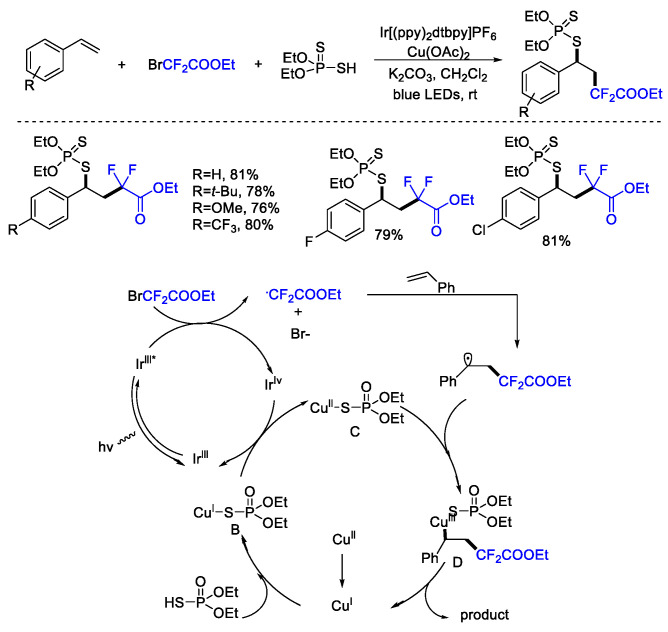
Photooxidation and copper-catalyzed fluoroalkyl thionation reaction.

In the past few years, great progress on photocatalysis has been made in the field of organic chemistry [62,63,64,65,66,67,68,69,70,71,72,73] since the pioneer works carried out by MacMillan et al. [74] and Yoon et al. [75] in 2008. In the presence of visible light, a difluoroalkylation reaction could proceed smoothly using RCF_2_X as an important synthon. The reduction of RCF_2_X by the photocatalyst via oxidative quenching was one of the most common methods to form the RCF_2_ radical. A photooxidation and copper-catalyzed fluoroalkyl thionation reaction of activated and unactivated olefins with a free-radical relay mechanism was reported by Cao and coworkers (Scheme 23) [76]. By using fluorohaloalkanes as free-radical precursors, and P(O)SH or P(S)SH compounds as coupling agents, various fluorine-substituted S-alkyl thiophosphate and dithiophosphorus esters can be obtained easily under mild conditions. In addition, this reaction strategy can be successfully used for the later functionalization of bioactive molecules.

**Scheme 24:**
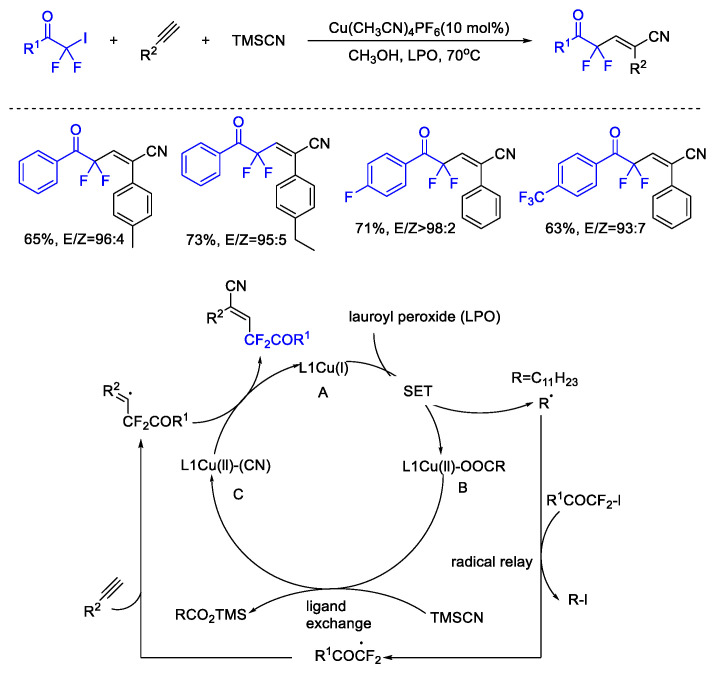
Synthesis of difluoroacyl-substituted nitriles catalyzed by copper.

Cu(I) catalyzed the three component reaction of 2-iodo-2,2-difluoroacetophenone, acetynes and TMSCN, which was presented by Wu’s group (Scheme 24) [77]. This reaction provides a simple strategy for the synthesis of difluoroacyl-substituted nitriles, which may be a potentially useful fluoro-organic intermediate for further conversion in drug discovery. The method displayed a wide substrate range and good stereoselectivity. The preliminary mechanism studies showed that a Cu(II)/Cu(I)-mediated free-radical process might be possible for this cyanodifluoroalkylation reaction.

**Scheme 25:**
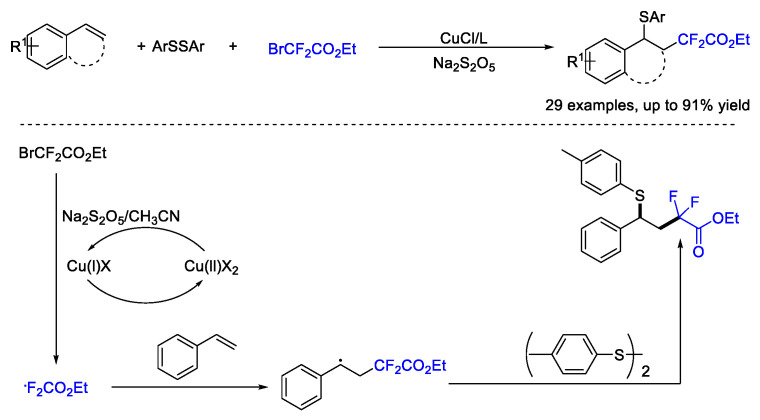
The difluoroalkylation of olefins catalyzed by Cu/Na_2_S_2_O_5_ system.

A new and simple method for the difluoroalkylation of olefins mediated by the Cu/Na_2_S_2_O_5_ system was reported by Zhang’s group (Scheme 25) [78]. The reaction was carried out under mild conditions using readily available ingredients, where C-C and C-S bonds were successfully constructed simultaneously. The reaction shows the characteristics of a wide substrate range of olefins and disulfides, good functional group tolerance, and high selectivity. A Cu(II)/Cu(I)-mediated radical mechanism was possible for this reaction. In this catalytic cycle, the Cu(I) species is obtained via the reduction in the Cu(II) species by a solvent (CH_3_CN) or Na_2_S_2_O_5_.

**Scheme 26:**
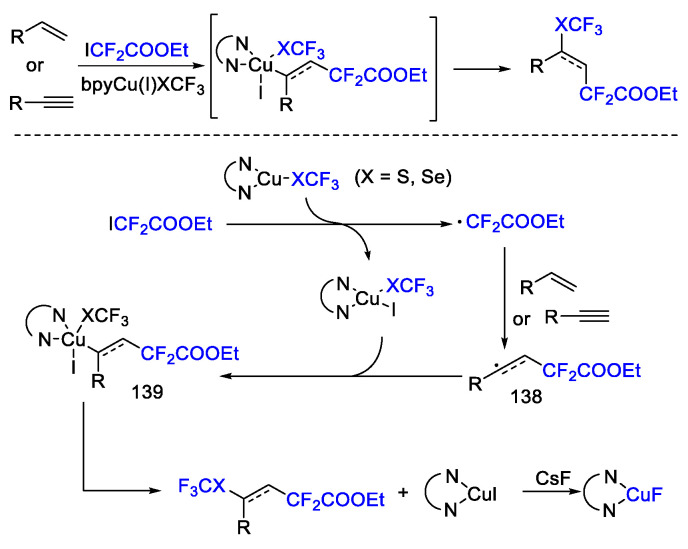
The copper-mediated difluoroalkylation reaction of alkynes with ethyl iododifluoroacetate.

In 2018, Liang and coworkers showed that air-stable SCF_3_ and SeCF_3_ reagents could serve as free-radical initiators of ethyl iododifluoroacetate via a reductive reaction (Scheme 26) [79]. With the assistance of air-stable SCF_3_ and SeCF_3_ reagents, the difluoroalkylation reaction between alkynes and the ethyl iododifluoroacetate proceeded efficiently. Successful avoidance of β-proton elimination was achieved. Besides alkynes, alkenes could also react smoothly in this reaction. The Cu(I)-Cu(II)-Cu(III) mechanism was proposed for this transformation.

## 4. Difluoroalkylation–Coupling Reactions

**Scheme 27:**
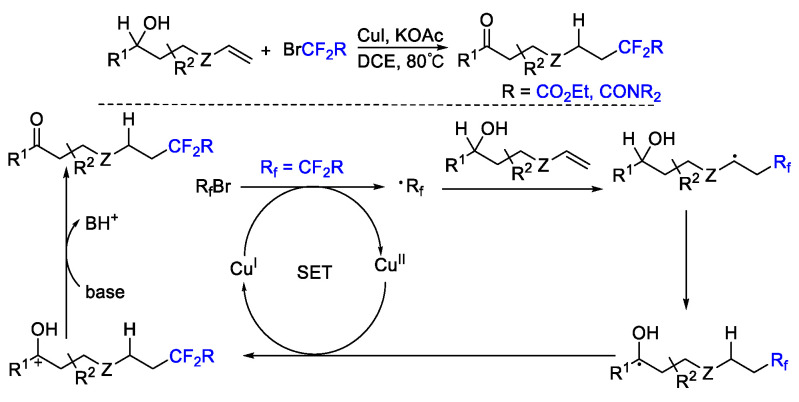
The Cu-catalyzed oxidation of alcohols with BrCF_2_CO_2_Et.

**Scheme 28:**
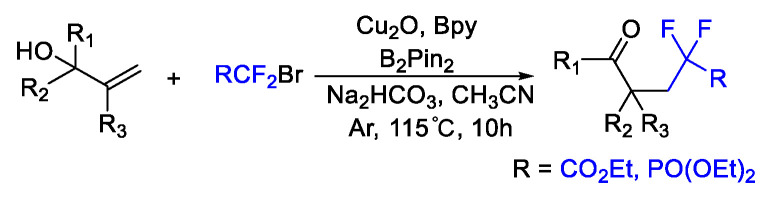
The Cu_2_O-catalyzed difluoromethylation of allylic alcohols.

In 2018, the Cu-catalyzed oxidation of alcohols with BrCF_2_CO_2_Et was realized by Cheng and coworkers, and various difluoroalkylated aldehydes or ketones were synthesized (Scheme 27) [80]. The reaction realized the catalytic oxidation of alcohol and the difluoroalkylation of olefins at the same time, which is an efficient and attractive organic synthesis method. Similarly, the radical difluoroalkylation of alkenes, intramolecular 1, 5- or 1, 6-HAT, SET oxidation and deprotonation reactions occurred in sequence to give the desired product. Xiong’s group also carried out the Cu_2_O-catalyzed phosphonyldifluoromethylation and ethoxycarbonyldifluoromethylation of allylic alcohols (Scheme 28) [81]. A radical 1,2-aryl migration was possible for the reaction mechanism.

**Scheme 29:**
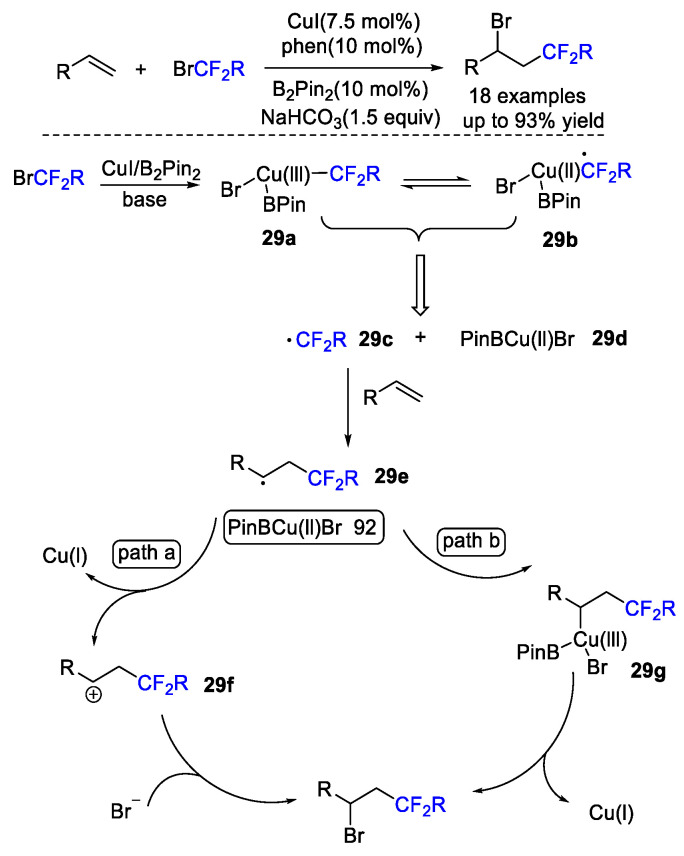
The copper-mediated difluoroacetylation of alkenes.

In 2018, the copper-catalyzed difluoroacetylation of alkenes with ethyl bromodifluoroacetate (BrCF_2_CO_2_Et) was presented by Zhu’s group (Scheme 29) [82]. With the help of a base, the reduction reaction between BrCF_2_CO_2_Et and the complex of Cu(I)/B_2_Pin_2_ occurred to provide CF_2_COOEt radical 29c and Cu(II) 29d. Then, carbon radical intermediate 29e was formed via the addition reaction between radical 29c and alkenes, and the final product was obtained via Cu(II)-mediated oxidation and bromide capture. Alternatively, reductive elimination of Cu(III) complex 29g, which was formed from the reaction of intermediate 29e with 29d, was also reasonable.

**Scheme 30:**
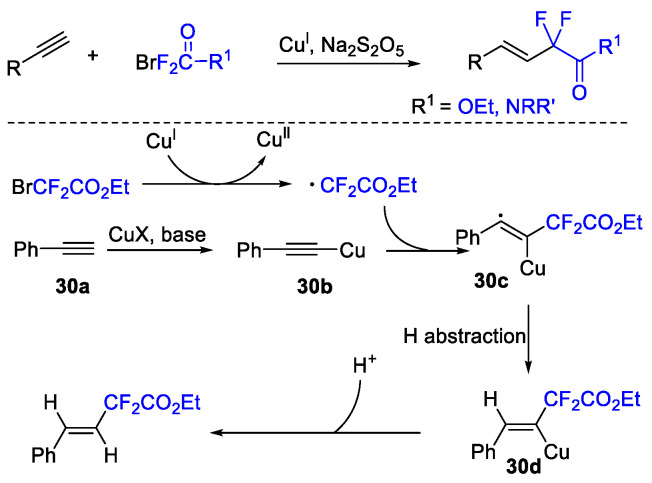
The copper-catalyzed hydrodifluoroalkylation of alkynes.

Wang and coworkers found that the hydrodifluoroalkylation reaction between alkynes and ethyl bromodifluoroacetate could be efficiently catalyzed by Cu (Scheme 30) [83]. Pyrosulfite should be used in this reaction as a reducing agent to inhibit the self-coupling of terminal alkynes. The SET reaction of BrCF_2_COOEt and Cu(I) could produce a CF_2_COOEt radical, which would be followed by an addition reaction with (phenylethynyl)copper 30b to form 30c. The final product was formed via hydrogen abstraction with solvents and protonation by moisture.

**Scheme 31:**
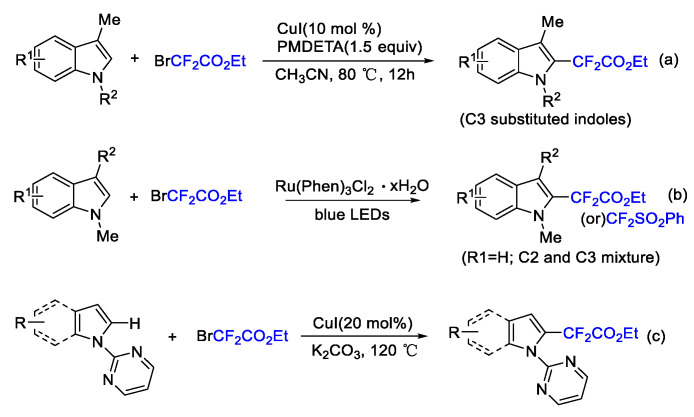
(**a**) Copper-catalyzed difluoroalkylation of indoles. (**b**) Ru-catalyzed difluoroalkylation of indoles. (**c**) Copper-catalyzed difluoroalkylation of indoles directed by pyrimidyl group.

The difluoroalkylation of heteroarenes, including indoles with BrCF_2_CO_2_R catalyzed by Cu (Scheme 31a) [84] and Ru (Scheme 31b) [85], was also realized by several groups. However, a mixture of C-2 and C-3 difluoroalkylation was obtained using these strategies. Therefore, C-3-substituted indoles are usually used in this reaction to obtain the product of selective C (2)-H difluoroalkylation. The C-2 difluoromethylation of indoles and pyrroles catalyzed by copper using BrCF_2_CO_2_Et was realized by Shi’s group. However, the pyrimidyl group, which acted as a direct group, should be present (Scheme 31c) [86].

**Scheme 32:**
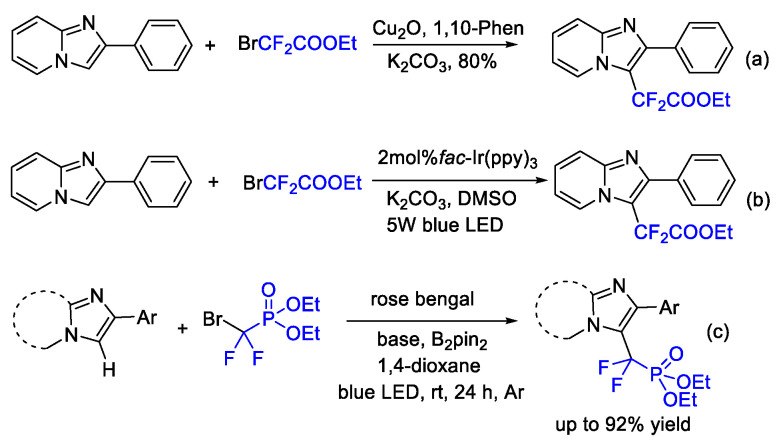
(**a**) Copper-catalyzed difluoroalkylation of imidazopyridines. (**b**) Ir-catalyzed difluoroalkylation of imidazopyridines. (**c**) Organophotoredox-catalyzed difluoromethylenephosphonation of imidazoheterocycles.

As a core structure, imidazole pyridine is widely present in many bioactive molecules and drugs [87,88]. Hajra and coworkers reported the copper-catalyzed difluoroalkylation of imidazopyridines with BrCF_2_CO_2_Et (Scheme 32a) [89]. Additionally, imidazo[2,1-b]thiazole and benzo[d]imidazo[2,1-b]thiazole can also react well in this reaction system. In 2017, Fu’s group demonstrated that the photocatalyst Ir could also promote this reaction (Scheme 32b) [90]. The first organophotoredox-catalyzed difluoromethylenephosphonation of imidazoheterocycles was realized by Hajra and coworkers (Scheme 32c) [91].

**Scheme 33:**
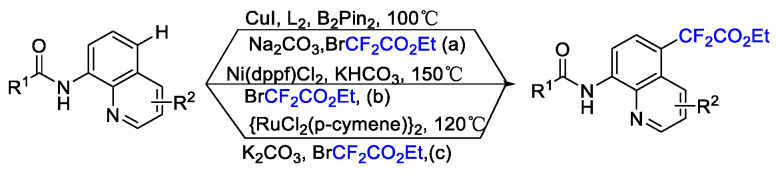
(**a**)The Cu-catalyzed difluoroalkylation of 8-aminoquinoline. (**b**) The Ni-catalyzed difluoroalkylation of 8-aminoquinoline. (**c**) The Ru-catalyzed difluoroalkylation of 8-aminoquinoline.

It was also proven that 8-aminoquinoline is capable of successfully performing difluoroalkylation with a difluoromethyl bromide reagent. The copper/B_2_pin_2_-mediated C-H ethoxycarbonyldifluoromethylation of 8-aminoquinoline scaffolds at the C5 position with functionalized difluoromethyl bromides and iodines was presented by Wu and coworkers (Scheme 33a) [92]. Ethoxycarbonyldifluoromethylation of naphthalenes at the C4 position was also realized in this system. Additionally, Ni- (Scheme 33b) [93] and Ru-mediated (Scheme 33c) [94] difluoromethylation of 8-aminoquinoline was reported separately by Wu, Wang and Zhao’s groups.

**Scheme 34:**
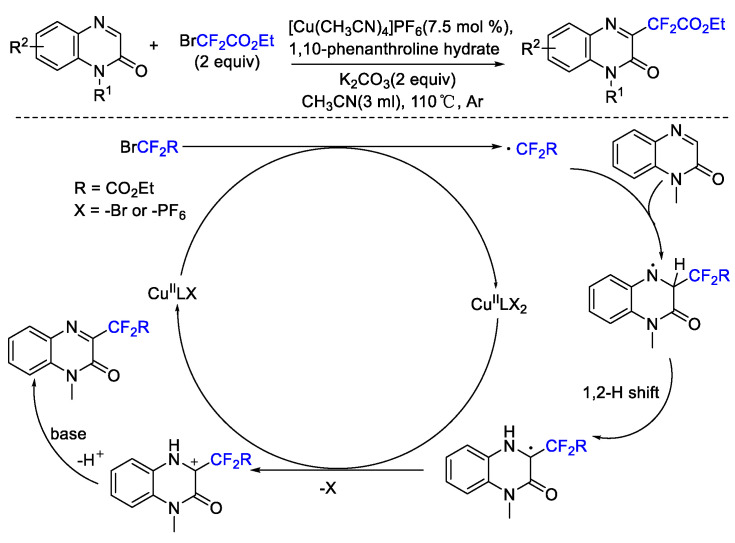
The copper-catalyzed direct C-3 difluoroacetylation of quinoxalinones.

Quinoxaline-2-ketone derivatives are widely found in bioactive molecules [71,95]. In 2019, the direct C-3 difluoroacetylation of quinoxalinones using ethyl bromodifluoroacetate catalyzed by copper was reported by Zhang’s group (Scheme 34) [96]. Control experiments were performed, implying that the CF_2_COOEt radical was involved in the catalytic cycle. Various difluoroacetylated quinoxalin-2(1H)-ones, which contained a wide range of functional groups, were isolated smoothly with moderate to excellent yields.

**Scheme 35:**
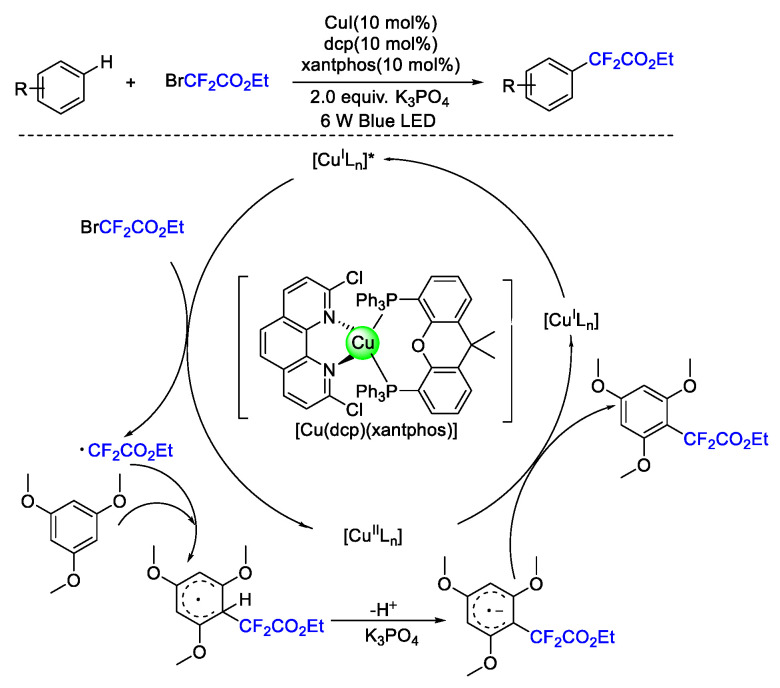
Visible-light-induced difluoroalkylation of arenes.

In 2019, the copper-catalyzed difluoroalkylation of arenes with difluoroalkylation reagents (BrCF_2_CO_2_Et or BrCF_2_CONR_1_R_2_) in the presence of visible light was realized by Liu and coworkers (Scheme 35) [97]. The in situ reaction of cuprous iodide, a triaryl phosphine ligand and an imine ligand formed a cuprous photocatalyst, playing a key role in this reaction. Difluoromethyl radicals that were changed via single-electron transfer from the excited photocatalyst into difluoroalkylation reagents were suggested in the difluoroalkylation reaction.

**Scheme 36:**
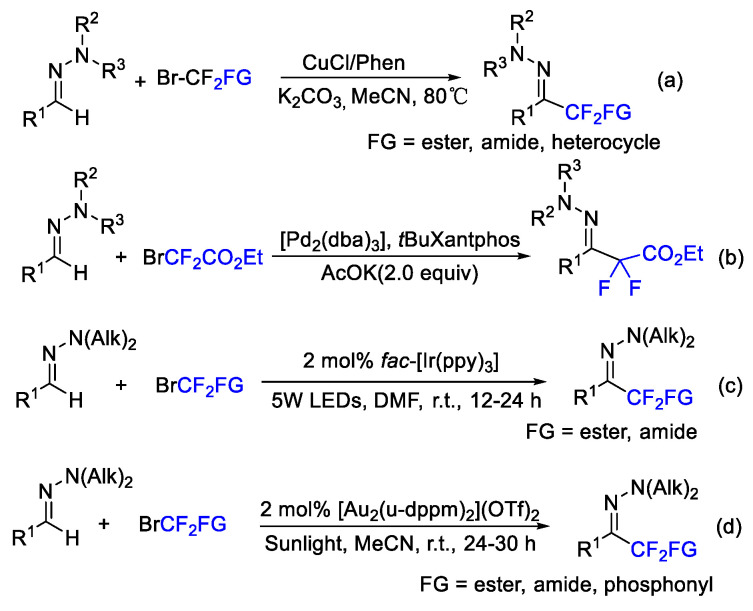
(**a**) Copper-catalyzed difluoroalkylation of hydrazones. (**b**) Pd-catalyzed difluoroalkylation of hydrazones. (**c**) Ir-catalyzed difluoroalkylation of hydrazones. (**d**) Au-catalyzed difluoroalkylation of hydrazones.

**Scheme 37:**
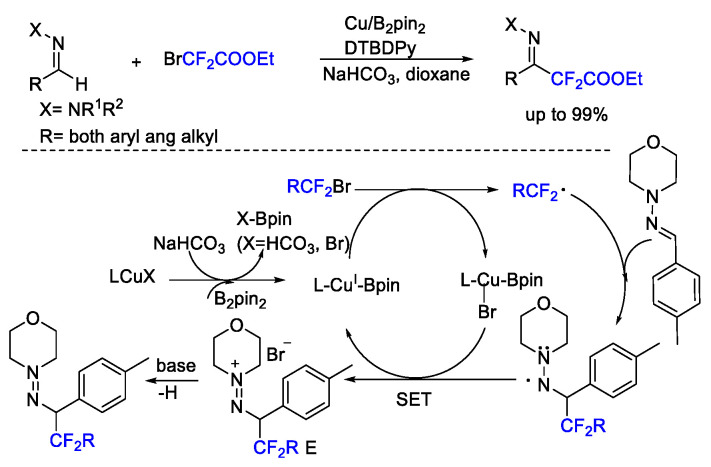
The Cu/B_2_pin_2_-catalyzed difluoroalkylation of hydrazones.

In 2016, the Cu(I)-catalyzed difluoroalkylation of hydrazones was realized by Monteiro’s group (Scheme 36a) [98]. Besides a copper catalyst, Pd_2_(dba)_3_ (Scheme 36b) [99], Ir (Scheme 36c) [100] and Au (Scheme 36d) [101] were also found to be effective in this reaction. The difluoroalkylation reaction between hydrazones and difluoroalkyl bromides catalyzed by Cu(II)/B_2_pin_2_ using diboron as a reductant was presented by Song and coworkers (Scheme 37) [102]. Both aliphatic and aromatic hydrazones can react smoothly in this reaction system. The SET reaction, which could provide a difluoroalkyl radical, was suggested in the catalytic cycle. Additionally, the diboron reagent played a key role in this reaction.

**Scheme 38:**
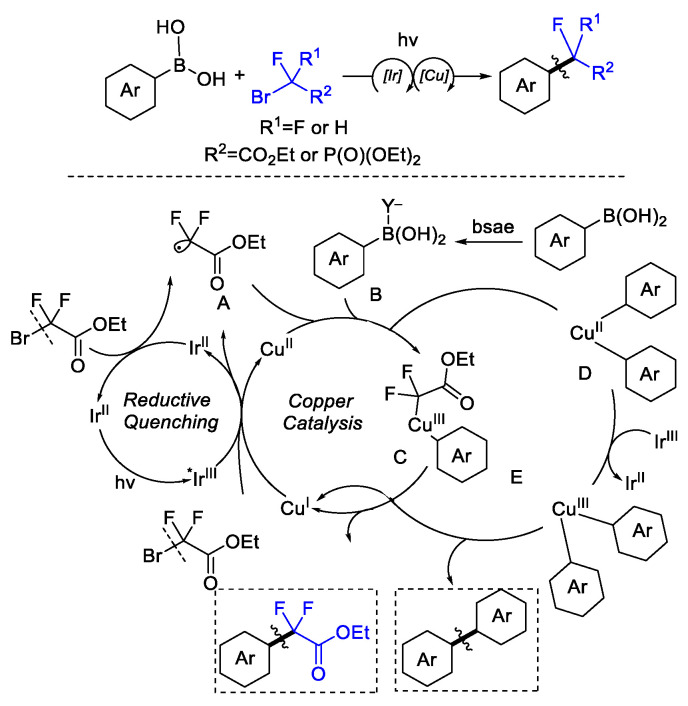
The Cu/B_2_pin_2_-catalyzed difluoroalkylation of hydrazones.

Molander and colleagues described a metal photoredox method for the preparation of fluoroalkyl aromatic hydrocarbons based on a bicatalytic Ir/Cu synergistic combination with boric acid (Scheme 38) [103]. Mild reaction conditions could tolerate a wide range of functional groups, such as aldehydes, free phenols, and N-BOC-protective amines. Mechanism studies support the process of photooxidation/copper dual catalysis. Reductive quenching of the excited photocatalyst Ir(III)* by Cu(I) was described in the catalytic cycle.

**Scheme 39:**
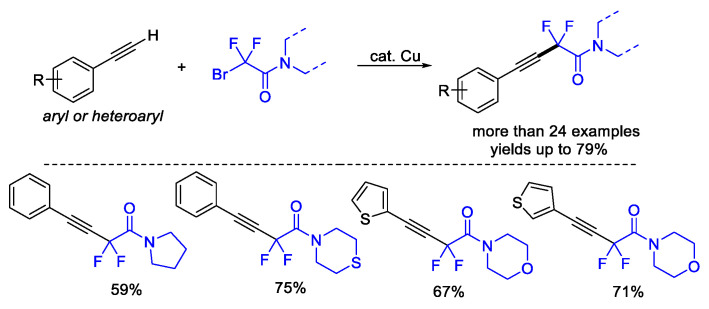
The Cu/B_2_pin_2_-catalyzed difluoroalkylation of hydrazones.

The free-radical alkylation of copper-catalyzed aryl acetylene with bromodifluoroamide was studied by Zhao’s group (Scheme 39) [104]. This reaction shows good functional group tolerance, and provides various substituted α-alkynyl-α, α-difluoroacetamides with medium to good yields. The potential for amplifying reactions and the derivatization of products also makes this method a great choice for practical applications. Preliminary mechanistic studies implied that catalytic systems may involve free-radical reaction pathways.

**Scheme 40:**
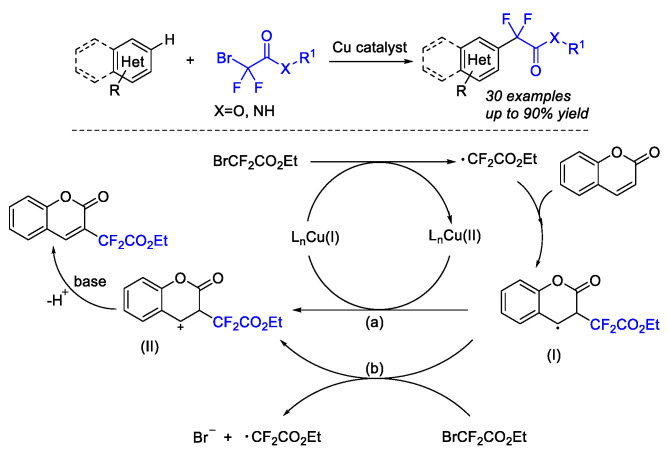
The selective C-H difluoroalkylation of coumarins catalyzed by copper.

An effective method for the selective C-H difluoroalkylation of coumarins catalyzed by copper is reported in [105] (Scheme 40). It is easy to obtain ethyl bromodifluoroacetate and N-phenylbromodifluoroacetamide. The reaction showed good functional group tolerance to coumarins and difluoroalkylation reagents, and several redox-sensitive substrates have been successfully applied to this difluoroalkylation reaction. This system can be further extended to other heterocyclic aromatic hydrocarbons, including furan, benzofuran, pyrrole, pyridone, chromone, indole and quinolinone. The in situ formation of fluoroalkyl radicals was proposed in this copper-catalyzed reaction.

**Scheme 41:**
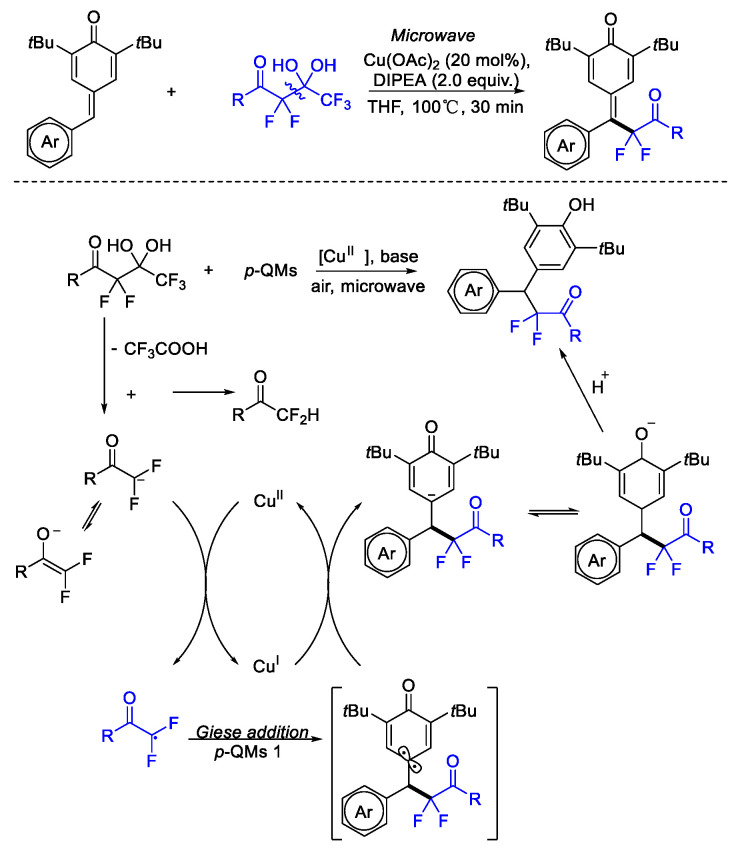
The microwave-assisted copper catalysis of α-difluorinated gem-diol.

Under microwave irradiation, the catalytic amount of oxidant Cu(OAc)_2_ was successfully applied to form transient difluoroalkyl radicals in situ for the first time (Scheme 41) [106]. The synthetic utility of this new method is also used to synthesize difluoroalkylated spirohexadienone, which is an important core structure in various natural products and pharmaceuticals. The Cu(II)/Cu(I)-mediated single-electron oxidation was described in the reaction mechanism.

## 5. Conclusions

Much progress has been made in the reaction of copper-catalyzed difluoroalkylation. In this review, the progress made with regard to copper-catalyzed difluoroalkylation reactions was summarized. At present, both visible-light-induced and metal-catalyzed difluoroalkylation were successfully realized. When alkenes or alkynes were subjected to these reactions, a radical addition to the carbon–carbon unsaturated bonds was usually proposed in the reaction mechanism; then, the corresponding products can be generated in different ways. RCF_2_X is the most commonly used difluoroalkylation reagent. In most cases, the reduction of RCF_2_X by copper to form the RCF_2_ radical was possible.

The C (sp3)-H bond has a high bond-dissociation energy, and lacks the “active” HOMO or LUMO orbital that interacts with the transition metal’s catalytic center, making it more difficult to functionalize the C (sp3)-H bond. The copper-catalyzed difluoroalkylation of the C(sp3)-H bond needs to be further researched. Considering green chemistry and cost-effectiveness, cheaper photocatalysts or even catalyst-free difluoroalkylation reactions are more desirable. There were only a few cases of visible-light-induced difluoroalkylation reactions catalyzed by copper reported in the literature. This issue is also worthy of further study. Asymmetric difluoroalkylation has not been reported yet. In addition, the multicomponent reaction when introducing the RCF_2_ group also needs further exploration. We expect that this review will highlight new ways for the development of difluoroalkylation reaction.
